# Round the Hospitals

**Published:** 1920-10-23

**Authors:** 


### ROUND THE HOSPITALS.

THE main topic at the meeting of the General Nursing Council held last week, and reported on page 87, appears to have been the scheme put forward by the College of Nursing for regulating the inclusion of nurses in the Hours of Employment Bill now before Parliament. This scheme, while safeguarding the interests of the entire profession, is of a nature to interfere but little with their work. To exclude private nurses from the provisions of the Bill would be to perpetuate the abuses under which they are now too often exploited for the benefit of grasping employers. To attempt to force a forty-eight-hour week on nurses in institutions would be resented by the very nurses it is desired to benefit, and might lead to the closing of many institutions. We trust that the statesmanlike scheme drawn up by the Council of the College may be adopted in its entirety by the Ministry of Health.

THE Secretary of State for India makes the following announcement: Nurses are required immediately for temporary service in the grade of staff nurse, with British troops in India. Applicants should be fully-trained nurses between the ages of twenty-seven and thirty-five. Midwifery qualifications are required, and the contract will include liability to serve in hospitals where the families of European troops receive treatment. Pay will be at the rate of Rs.250 per mensem. Engagement will be for six months, extensible at the option of the Government of India to one year. Selected candidates (if below the age of thirty-two on appointment) may be considered on termination of contract for such vacancies in the permanent service as may then exist. No gratuity is payable on completion of service, but free passage both ways is given. Outfit allowance of £20, or £25 in the case of those who have not previously served with a military nursing service, will be granted. Free quarters, fuel, light, and punkah-pullers are allowed in addition to pay. Inquiries should be addressed to the Secretary, Military Department, India Office, Whitehall, S.W. 1, and be clearly marked "Temporary Nurses" on the top left-hand corner of the envelope. Original certificates and testimonials should not be sent.

THE General Purposes Committee of the Lambeth Borough Council have bowed to the express wish of the majority of the Council, and, having reconsidered their recommendation of a month ago, have withdrawn it, and decided that Miss T. McHugh, a lady health visitor who has been married, should be allowed to continue in her office. The facts were reported in THE HOSPITAL on October 2, page 16. This is a distinct victory for the lady members of the Borough Council, especially for Mrs. Councillor La Chard, who was responsible for obtaining the support of her colleagues in getting the Committee to reconsider their decision that the time had arrived when Miss McHugh should be asked to resign her office in favour of a lady who was either unmarried or a widow. So far as Lambeth is concerned this decision disposes definitely of the question as to whether an officer, either a female sanitary inspector, health visitor, or tuberculosis nurse, shall have to resign when she gets married.

IT was possible to realise for the first time, on the recent anniversary of Edith Cavell's death, how much the nursing profession has gained by having a spot in London dedicated to her in the statue raised to her memory by the efforts of the *Daily Telegraph*. This beautiful memorial of the nurse, which gains more and more upon all who habitually pass by, was the spot to which those who cherish her ideals and revere her example naturally turned on the anniversary of her death. The co-operation of her two sisters, Mrs. Wainwright and Miss Scott Cavell, in the task of consolidating the Nurses' Homes of Rest which were the dream of her heart, should reassure anyone disposed to question the policy of the Council of the Edith Cavell Homes in holding a Flag Day for this purpose on the anniversary. We understand the results have been very satisfactory, while there is reason to hope the current of interest stirred will bring in additional sums in course of time.

THE memorial to Miss Florence Nightingale Shore, the cousin and god-child of Florence Nightingale, has not yet been fully subscribed, and it is much desired to secure more money to found the Nurses' Home with Children's Treatment Centre which has been projected at Hammersmith for this purpose. It will be remembered that Miss Shore was found murdered in the Hastings express last January and no clue to the murderer has ever been forthcoming. Her work in war and in peace was wholly devoted, and it is not only among her numerous friends that the wish has been manifested of commemorating her services. Subscriptions are received by the Hon. Treasurer, Councillor Marshall Hays, 22 St. Peter's Square, Hammersmith, W. 6.

THE Birmingham District Nursing Institution appears to be still going downhill. The Home at 94 Moseley Road is to be closed on November 1, and the nursing staff has been reduced by half. It cannot be doubted that this dwindling process will continue, unless checked by an immediate forward policy, until the Society becomes extinct. The proposal now adopted to charge a fee for visits should long ago have been put in force, and the fee should be adequate. Times have changed, and working-class incomes have so appreciably increased that nursing on a purely eleemosynary basis is doomed. The attempt to provide free nursing for all the sick in Birmingham has proved an impossible task. But not for that reason need the Birmingham District Nursing Association come to an end.

**ROUND THE HOSPITALS**—(*continued*).

A thorough reorganisation is called for, and when that has taken place the task of wiping off the debt of some £3,000 which now hampers its operations should present no difficulties.

At Lincoln, under the Lady Superintendent, Miss Somerset, the payment system has been recently introduced into the Nursing Association, and the results are proving highly satisfactory. Since last May 137 cases have been nursed, and the fees received amount to £76 18s. from eighty-eight paying cases. It has been decided to raise the family income figures on householders' cards by 10s. all round. The question of providing nurses for the districts to be added to the city in November is under consideration, and the nursing needs of the whole district appear to be well understood.

The training centre for midwives and village nurses so long desired by the Northumberland County Nursing Association is beginning to materialise. There are over eighty district nursing associations affiliated to the parent, with 120 nurses at work, and the task of keeping all these districts supplied becomes increasingly difficult owing to the lack of any training facilities. The Association has now obtained a site at Willingdon for the erection of a block of Army huts suitable for a nurses' hostel, from which it is proposed to carry out the training of pupils under fully-qualified supervision. The cost of initiating the scheme is estimated at no more than £2,000, a sum which provides for the purchase, removal, re-erection, completion, and furnishing of the huts, and of this £500 has been already subscribed.

The Lincoln Guardians are still halting between two opinions in respect of their infirmary. The Labour Guardians are pressing for compliance with the demands of the Ministry of Health, and maintain that the old infirmary is "not fit to die in." One of the clerical members of the Board is heard asserting in reply that though out of date the infirmary is as clean as a new pin, and that he himself should not be afraid to die in it. Cleanliness and care are present, and a ministry of angels in the nurses, who deserve all praise for the care they show to the dying poor. These are higher matters undoubtedly than mere "up-to-dateness," but we are not convinced that they would not flourish all the more blithely in a building better suited to the requirements of the sick.

The Royal Bucks Hospital benefits to the extent of over £2,000 as the result of an able rally on its behalf undertaken by the Mayor of Aylesbury, Mr. J. Robinson, who set himself to the task of raising £1,200 in a fortnight. The means adopted were the circulation of appeals to all parts of the country, supplemented by a country fair, a giant jumble sale, and two concerts. The excellent result testifies to the value of setting about such enterprises with determination and energy. To stimulate the spirit of giving, all the promoters must be fired with enthusiasm, and when that has been achieved everyone else psychologically follows suit. Such a short and sharp effort is better for the hospital and better for its supporters than a whole twelvemonth of broken-hearted appeals issued with a conviction that nothing can possibly result from them.

Deplorable accounts have been coming through recently about the Petrograd hospitals, but we are glad to note that the Swedish Red Cross Society has recently sent delegates to report on this matter and that after a thorough investigation these authorities have found the conditions not quite so bad as they had feared. There is very little serious disease except dysentery. The hospitals are open to all without regard to social standing. But there is a great scarcity of medicines, of surgical instruments and of anæsthetics, a circumstance which cannot fail to hamper the work of the medical staff to a deplorable extent. Large supplies of hospital requisites are being furnished by the Swedish Red Cross through the medium of a representative of the "Save the Children" fund, who will be accompanied by a Swedish doctor and an interpreter, and will be furnished with special supplies of food and medicines for the children. Miss Elsa Brandström, who has lately returned from five years' work among the prison camps of Russia and Siberia, is making an earnest appeal for money and clothing.

The provision of midwives through philanthropic agencies is being threatened in more than one direction. At Plymouth we learn that owing to the shortage of midwives it is proposed by the Three Towns Nursing Association to discontinue its nursing services for the Corporation forthwith, and the Maternity and Child Welfare Committee has asked the Medical Officer of Health to submit a scheme for continuing the service. At Bradford, where the midwifery service is already in the hands of the Health Committee of the Corporation, an acute shortage of midwives prevails, no applications are being received for midwifery appointments in answer to advertisements, and the Corporation has decided to raise the present salary of £80 plus war bonus to £100. The fruits of the long under-payment for midwifery services are now being reaped, and we have by no means reached a stable equilibrium as regards salaries.

We are glad to note that at a recent meeting of the Board of Management of the South London Hospital for Women a resolution was passed sanctioning increases in the scale of nursing salaries as follows:—Sisters: Commencing salary £60, rising by £5 to £70. Staff nurses (certificated): Commencing salary £50, rising by £2 to £56. Probationers: First year £24, second year £28, third year £32.

